# Congenital lower urinary tract obstruction with spontaneous fetal bladder rupture due to posterior urethral valves: a case report

**DOI:** 10.1186/s13256-023-04163-x

**Published:** 2023-10-25

**Authors:** Max Adriaenssens, Veerle De Boe

**Affiliations:** Department of Urology, Universitair Ziekenhuis Brussel, Brussels, Belgium

**Keywords:** Lower urinary tract obstruction, Posterior urethral valves, Bladder rupture, Congenital diseases

## Abstract

**Background:**

Congenital lower urinary tract obstruction (LUTO) is a rare but significant condition affecting fetal urinary tract development. LUTO has a range of etiologies, with posterior urethral valves (PUV) being the most common cause. The prenatal diagnosis of LUTO plays a crucial role in recognizing the condition and guiding management decisions. Prenatal ultrasound serves as the primary tool for identifying LUTO, with key findings including megacystis, bladder wall thickening, oligohydramnios, hydronephrosis, and the 'keyhole sign' indicating dilatation of the posterior urethra. We present a case of congenital LUTO with a rare complication of spontaneous fetal bladder rupture and urinary ascites, treated by peritoneo-amniotic shunt placement.

**Case presentation:**

A 27-year-old pregnant Caucasian women was referred at 28 weeks of pregnancy due to the presence of megacystis and bilateral hydronephrosis on routine ultrasound and suspicion of LUTO. Repeat ultrasound at 29 weeks showed significant fetal ascites, oligohydramnios and resolution of megacystis and hydronephrosis, after which diagnosis of spontaneous bladder rupture was made. Despite ascites aspiration and amnio-infusion, there was persistent ascites and oligohydramnios. A peritoneo-amniotic shunt was placed with resolution of ascites and normalization of the amniotic fluid volume. At 35 weeks, relapse of the megacystis was observed with bilateral pyelectasis and oligohydramnios, possibly due to healing of the bladder rupture, after which elective cesarean section was planned. Cystography confirmed spontaneous healing of the bladder rupture and the presence of posterior urethral valves, which were resected in the neonatal period with cold knife incision. Total follow-up of 8 years continued to show positive ultrasonographic results and good renal function, but the child suffers from bladder dysfunction, manifesting as overactive bladder disease.

**Conclusions:**

LUTO might lead to important renal dysfunction and pulmonary hypoplasia in case of increasing disease severity. Spontaneous bladder rupture might improve renal prognosis, acting as a pop-off mechanism by decompression of the urinary tract. However, fetal bladder rupture is rare and only few cases have been reported. Prenatal intervention can be considered for moderate or severe LUTO, but the benefit for long-term outcome remains uncertain and further studies are needed.

## Background

Congenital lower urinary tract obstruction (LUTO) is a rare but significant condition affecting fetal urinary tract development. It is characterized by the obstruction of urine flow at various levels, leading to the accumulation of urine within the urinary tract and subsequent consequences for fetal renal function and lung development. LUTO has a range of etiologies, with posterior urethral valves (PUV) being the most common cause, followed by urethral atresia and Prune-belly syndrome.

The prenatal diagnosis of LUTO plays a crucial role in recognizing the condition and guiding management decisions. Prenatal ultrasound serves as the primary tool for identifying LUTO, with key findings including megacystis, bladder wall thickening, oligohydramnios, hydronephrosis, and the ‘keyhole sign’ indicating dilatation of the posterior urethra.

We present a case of congenital LUTO with a rare complication of spontaneous fetal bladder rupture and urinary ascites, treated by peritoneo-amniotic shunt placement. We report the pre- and post-natal management of our case followed by an overview of the literature.

## Case presentation

A 27-year-old pregnant Caucasian woman (gravida 2, para 1) was referred to our center at 28 weeks of pregnancy due to the presence of megacystis and bilateral hydronephrosis on routine ultrasound, which suspected LUTO. At this time, the amniotic fluid index (AFI), lung status and the presence of a clear “keyhole sign” due to posterior urethral dilatation were not mentioned in the report. Previous ultrasound examinations at 20 and 24 weeks' gestational age were completely normal. The family history revealed a sister with a horseshoe kidney and a nephew with unilateral pyelectasis. A repeat ultrasound at our center at 29 (6/7d) weeks of gestation showed significant fetal ascites with an elevated diaphragm and thoracic compression. Oligohydramnios was present with an AFI of 2.7 cm. Urinary tract evaluation showed significant bladder wall thickening (6.2 mm) but the resolution of previously observed megacystis and hydronephrosis, suggesting a spontaneous bladder rupture with the onset of urinary ascites. The renal parenchyma appeared normal. The patient was admitted for cardiotocography (CTG) fetal monitoring, initiation of corticosteroid therapy for fetal pulmonary maturation, and further work-up.

**Fig. 1:**
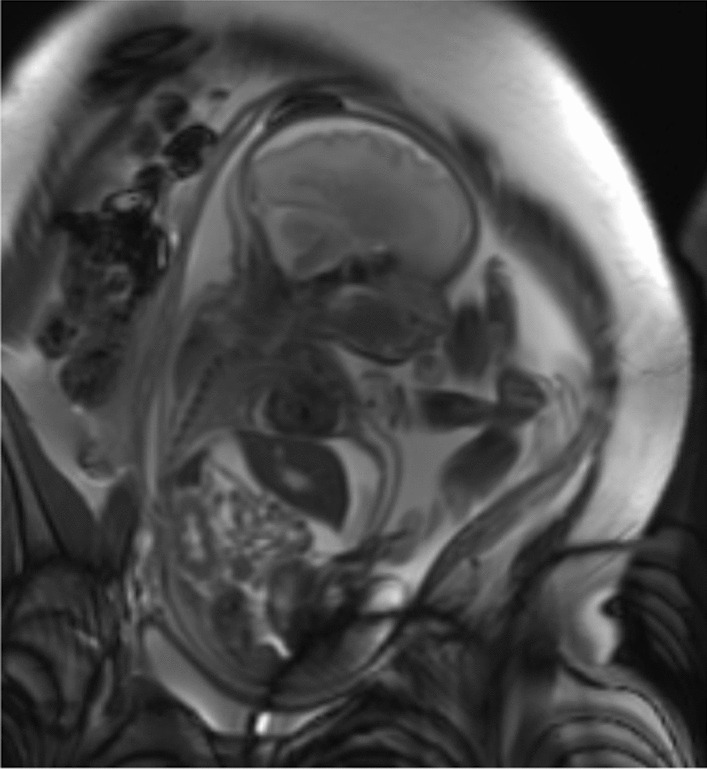
MRI T2 image showing fetal urinary ascites compressing the thoracic cavity, but no signs of pulmonary hypoplasia and normal kidneys

Initially, a minimal invasive approach was chosen to avoid vesico-amniotic shunt (VAS) placement, which has a relatively high complication rate. Percutaneous ultrasound-guided fetal ascites puncture was performed with the aspiration of 420 ml of fluid, followed by amnio-infusion of 250 ml. Analysis of the urinary ascites showed a normal male karyotype and a β-2-microglobulin value of 2.1 mg/L, indicating good renal prognosis. MRI confirmed previous ultrasonographic findings of spontaneous bladder rupture with fetal urinary ascites compressing the thoracic cavity (Fig. 1). There were no signs of pulmonary hypoplasia, and the kidneys appeared normal. Despite ascites aspiration, a repeat ultrasound at 30 (4/7d) weeks showed an increasing fetal abdominal circumference with persistent ascites and oligohydramnios. Fetal Doppler ultrasound showed a decreased venous return with presence of pulsatile flow in the inferior vena cava and a continuous elevated fetal heart rate between 150 and 170 beats per minute, leading to cardiac decompensation. Due to these cardiovascular changes, the decision was made for repeat ascites drainage and peritoneo-amniotic shunt placement. Because of the spontaneous rupture, bladder filling was insufficient for classic VAS placement. Due to the lack of experience with this procedure in our hospital, the intervention was performed at a tertiary center fetal surgery department. Fetal ultrasonography one day post-operatively showed no residual ascites, correct stent position, absence of hydronephrosis, and normalization of the AFI to 10.8 cm. The patient was transferred back to our center for further follow-up and perinatal care.

**Fig. 2:**
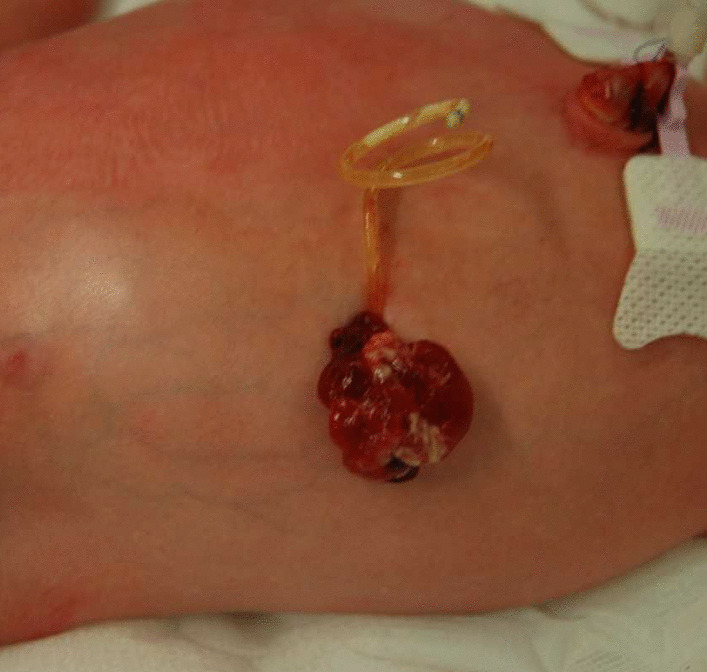
Prolapse of intra-abdominal tissue next to peritoneo-amniotic shunt, which was shown to be omentum on explorative surgery

At 34 (6/7d) weeks, relapse of the megacystis was observed with bilateral pyelectasis and oligohydramnios (AFI 2.4 cm), possibly due to healing of the bladder rupture, after which elective cesarean section was planned. The male infant weighed 2370 g with Apgar scores of 9 and 10 at 1 and 5 minute, respectively, and there was no need for supportive measures. A 6 Fr transurethral bladder catheter was placed with drainage of 90 ml of urine, and amoxicillin uroprophylaxis was initiated. Inspection of the peritoneo-amniotic shunt showed prolapse of intra-abdominal tissue (Fig. 2). Exploratory surgery showed the tissue to be a part of omentum, which was resected. The shunt was removed, and the abdominal wall defect was closed.

**Fig. 3:**
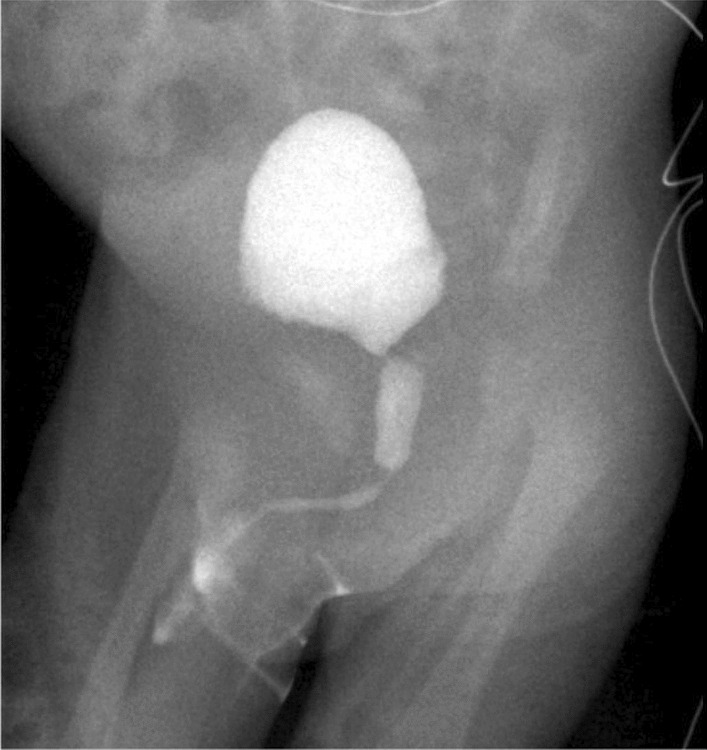
Postnatal cystography showing posterior urethral valve and resolution of bladder rupture

**Fig. 4:**
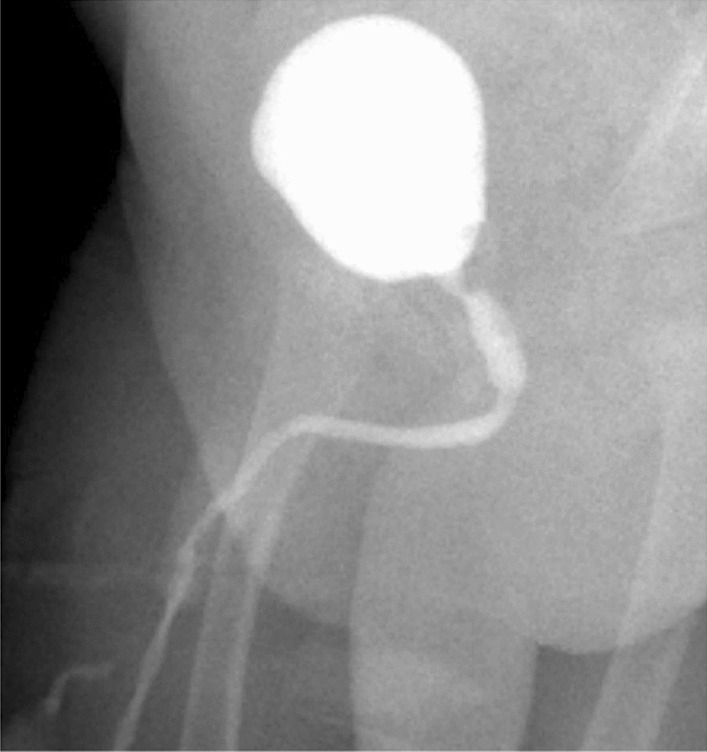
Post-operative repeat cystography at 12 months showing no residual posterior urethral valve

Cystography performed 1 week after birth confirmed the diagnosis of PUV, and also confirmed resolution of the bladder rupture and a right-sided grade I vesico-ureteral reflux (VUR) (Fig. 3). An 8 Fr transurethral catheter was left in place which was changed for a 10 Fr catheter one week later to allow cystoscopy and PUV resection with a 9.5 Fr resectoscope at the age of one month, using cold knife incision at the 5 and 7 o’clock positions. The bladder catheter was removed on the first post-operative day, after which there was good micturition and absence of post-void residual volume (PVR). Ultrasonographic follow-up showed resolution of hydronephrosis and gradual decrease of bladder wall thickening over the following months. At 3 months of age, uroprophylaxis was changed to nitrofurantoin. Dimercaptosuccinic acid (DMSA) scan and Cr-ethylenediaminetetraaceticacid (EDTA) study at 6 months showed normal kidneys with symmetrical function and good absolute renal clearance of 71.9 ml/minute/1.73 m^2^. At 12 months, in consultation with the child’s parents, a repeat cystography was performed instead of a second look cystoscopy to avoid general anesthesia and to evaluate both the presence of residual PUV and VUR. The exam showed no residual PUV and there was minimal residual gr I VUR on the right side (Fig. 4). Therefore, uroprophylaxis was terminated.

Six-monthly follow-up with urinary tract ultrasonography and urinary sample was normal, and a repeat Cr-EDTA study at three years showed normal renal clearance of 151 ml/minute/1.73 m^2^. We have a total follow-up of 8 years, which has continued to show normal ultrasonographic findings and renal function. However, we report the presence of bladder dysfunction, manifesting as overactive bladder with daytime urinary incontinence, low bladder capacity and nocturnal enuresis. Standard urotherapy was initiated with limited result. Transcutaneous electrical nerve stimulation (TENS) was started as a second line treatment since the parents preferred to avoid medical treatment. There was a positive evolution of symptoms with increasement of the bladder capacity to approximately 300 ml, however with persisting urgency and urgency-incontinence. Anticholinergic therapy (oxybutynin, 2.5 mg two times daily) showed better results with resolution of the urinary incontinence. Currently, anticholinergic treatment is still ongoing and dose de-escalation is planned.

## Discussion and conclusions

Congenital LUTO represents a total prevalence of 3.34 (2.95–3.72) per 10,000 pregnancies [1]. The most common causes are posterior urethral valves (PUV) in 63% of cases followed by urethral atresia and Prune-belly syndrome. Less common causes include anterior urethral valves, anterior urethral diverticulum, congenital megalourethra and obstructing ureterocele [2].

The primary tool for diagnosing LUTO is prenatal ultrasound. Bladder identification on prenatal ultrasound corresponds with the start of fetal urine production which approximately starts at 10 weeks of gestation [3]. LUTO is usually diagnosed before 28 weeks of gestational age and low amniotic fluid levels secondary to LUTO might be detected on prenatal ultrasound starting from 16 weeks gestation. Possible signs on ultrasound that might suggest LUTO include the presence of megacystis, bladder wall thickening, oligohydramnios, hydronephrosis and the ‘keyhole sign’ due to dilatation of the posterior urethra. The classic triad of ultrasound findings used as indicator for LUTO is typically described as megacystis, ‘keyhole sign’ and hydronephrosis.

Unlike others, Bernardes *et al.* stated that increased bladder wall thickness and bladder dilation were strong predictors of PUV diagnosis, but the ‘keyhole sign’ did not predict the diagnosis [4]. In order to differentiate LUTO from non-obstructive causes of megacystis, Fontanella *et al.* designed a scoring system improving diagnostic accuracy of LUTO [5]. Variables used in the scoring system included severity of megacystis, bilateral ureteral diameters, presence of oligo- or anhydramnios, male fetal sex and referral at < 28 weeks of gestation. A score of ≥ 9.5 indicated a risk of LUTO of 96%. According to the ERKNet CAKUT-Obstructive Uropathy Work Group the use of the antero-posterior diameter of the renal pelvis is the most reliable parameter for suspecting obstructive uropathies and for suspecting prenatal LUTO in the presence of fetal megacystis. They also suggest that the risk of prenatal and neonatal death depends on the presence of oligo- or anhydramnios before 20 weeks gestation [6].

LUTO might lead to renal dysfunction and lung hypoplasia in case of oligo- or anhydramnios. In order to select which cases might need prenatal intervention it is important to understand the natural history of LUTO according to disease severity. Johnson *et al.* studied the natural course of LUTO in cases with normal amniotic fluid values at midgestational age and tried to define which prenatal disease parameters influence fetal outcome [7]. Study results showed that fetuses with normal amniotic fluid had low perinatal mortality and that surviving fetuses had a minimal risk of neurodevelopmental, respiratory and musculoskeletal morbidity. The majority of cases with favorable postnatal renal function showed stable function during the first two years of life, however, still 1/3 of cases required renal replacing therapy afterwards. Fetuses with low-normal amniotic fluid levels at 24 weeks gestation had an increased risk of renal dysfunction [7].

Fontanella *et al.* studied antenatal factors associated with perinatal mortality and postnatal renal function in 261 fetuses suspected with LUTO which were managed conservatively. They developed a staging system according to bladder volume and gestational age at oligo- or anhydramnios presentation, dividing LUTO into mild (stage I), moderate (stage II) and severe (stage III) disease groups. Worse stage was shown to have worse outcome with increased perinatal mortality and renal impairment. Early gestational age at oligo- or anhydramnios presentation and high bladder volume were associated with high morbidity and mortality [8]. Several comparable staging systems have been designed using some different parameters. For example, Ruano *et al.* proposed a LUTO classification in which besides amniotic fluid level, fetal urinary biochemistry and ultrasonographic characteristics of the kidneys like echogenicity, dysplasia and cortical cysts were also included [9]. In our experience, the renal ultrasound findings in particular should be taken into account and might have an important role in defining renal prognosis and disease management.

The three stage system of disease severity is currently used to guide decision making in prenatal interventions for LUTO. When considering fetal intervention it is important to try to avoid unnecessary treatment in cases unlikely to benefit due to severity of the disease as well as avoiding complications in those who are likely to survive and have good prognosis with conservative management. In stage I LUTO, fetal intervention is not indicated, unless there is progression to stage II disease where it might be considered. Prenatal therapy is mostly indicated in stage II disease to prevent pulmonary hypoplasia and severe renal impairment. Therapy for stage III disease can be considered but only to aid in lung development since renal damage at this stage is irreversible [9].

Potential prenatal interventional therapies for LUTO include repeat vesicocentesis, fetal vesico-amniotic shunt placement and fetal cystoscopy, as well as termination of pregnancy. The most widely used treatment is VAS, since this technique is technically easier and less invasive than fetal cystoscopy. However, the complication rate is quite high with stent migration, stent blockage and premature rupture of the membranes being the most common. The PLUTO trial is the only available randomized study evaluating the outcome of VAS. In the trial, overall perinatal survival seemed to be higher in the VAS treated fetuses. However, the results suggested that the renal function prognosis in these newborns was poor irrespective of whether or not VAS was performed [10]. Unfortunately, the findings of the study were limited due to low patient recruitment and results should be interpreted with this limitation in mind.

In our case we described a late presentation of LUTO at 28 weeks of gestation, supporting favorable disease progression. Fast progression to spontaneous bladder rupture might have improved renal outcome due to relief of pressure on the upper urinary tract. Other pop-off mechanisms, decompressing the obstructed urinary tract are known like formation of bladder diverticulum, unilateral high grade reflux disease and renal forniceal rupture with urinoma formation. However, fetal bladder rupture is rare and there are few reported cases with various causes like PUV, anterior urethral valves (AUV), meningomyelocele, spina bifida and iatrogenic due to maternal high doses of opioids for sedative purposes [11, 12]. With its late LUTO presentation, the fetus might have had good prognosis with a conservative management. However, in our case treatment was necessary due to the persisting ascites with thoracic and cardio-venous compression. Prenatal treatment for LUTO is still considered valuable but further studies are needed to identify predictors and modifiable risk factors associated with disease progression and severity and to evaluate the long-term outcomes of selective therapy based on disease severity.

## Data Availability

Data sharing is not applicable to this article as no datasets were generated or analysed during the current study.

## Abbreviations

LUTO: Lower urinary tract obstruction
PUV: Posterior urethral valves
AFI: Amniotic fluid index
CTG: Cardiotocography
VUR: Vesico-urethral reflux
PVR: Post-void residual volume
DMSA: Dimercaptosuccinic acid
EDTA: Ethylenediaminetetraaceticacid
TENS: Transcutaneous electrical nerve stimulation
VAS: Vesico-amniotic shunt
